# Evaluation of Direct and Cell-Mediated Lactoferrin Gene Therapy for the Maxillofacial Area Abscesses in Rats

**DOI:** 10.3390/pharmaceutics13010058

**Published:** 2021-01-04

**Authors:** Elima Agatieva, Said Ksembaev, Mikhail Sokolov, Vage Markosyan, Ilnaz Gazizov, Dmitry Tsyplakov, Maxim Shmarov, Irina Tutykhina, Boris Naroditsky, Denis Logunov, Oskar Pozdeev, Lidiya Morozova, Kamilya Yapparova, Rustem Islamov

**Affiliations:** 1 Department of Maxillofacial Surgery and Surgical Dentistry, Kazan State Medical University, 420012 Kazan, Russia; elly87@mail.ru (E.A.); ksesa@mail.ru (S.K.); 2 Department of Operative Surgery and Topographic Anatomy, Kazan State Medical University, 420012 Kazan, Russia; supermihon@yandex.ru (M.S.); vage6993@mail.ru (V.M.); ilnazaziz@mail.ru (I.G.); 3 Department of Pathology, Kazan State Medical University, 420012 Kazan, Russia; Dr.AllaKazan@yandex.ru; 4 The National Research Center for Epidemiology and Microbiology Named after Honorary Academician N.F. Gamaleya of the Ministry of Health of the Russian Federation, 123098 Moscow, Russia; mmshmarov@gmail.com (M.S.); their@yandex.ru (I.T.); bsnar1941@yahoo.com (B.N.); ldenisy@yahoo.com (D.L.); 5 Department of Microbiology, Kazan State Medical Academy, 420012 Kazan, Russia; pozdeevoskar@rambler.ru (O.P.); lidiya.morozova.1957@mail.ru (L.M.); 6 Department of Medical Biology and Genetics, Kazan State Medical University, 420012 Kazan, Russia; yap.kamila@gmail.com

**Keywords:** maxillofacial area phlegmon, rat, gene therapy, adenoviral vector, human umbilical cord blood mononuclear cell, lactoferrin gene, wound healing, lymph node

## Abstract

Resistance to antibacterial therapy requires the discovery of new methods for the treatment of infectious diseases. Lactoferrin (LTF) is a well-known naïve first-line defense protein. In the present study, we suggested the use of an adenoviral vector (Ad5) carrying the human gene encoding LTF for direct and cell-mediated gene therapy of maxillofacial area phlegmon in rats. Abscesses were developed by injection of the purulent peritoneal exudate in the molar region of the medial surface of the mandible. At 3–4 days after phlegmon maturation, all rats received ceftriaxone and afterward were subcutaneously injected around the phlegmon with: (1) Ad5 carrying reporter *gfp* gene encoding green fluorescent protein (Ad5-GFP control group), (2) Ad5 carrying *LTF* gene (Ad5-LTF group), (3) human umbilical cord blood mononuclear cells (UCBC) transduced with Ad5-GFP (UCBC + Ad5-GFP group), and (4) UCBC transduced with Ad5-LTF (UCBC + Ad5-LTF group). Control rats developed symptoms considered to be related to systemic inflammation and were euthanized at 4–5 days from the beginning of the treatment. Rats from therapeutic groups demonstrated wound healing and recovery from the fifth to seventh day based on the type of therapy. Histological investigation of cervical lymph nodes revealed purulent lymphadenitis in control rats and activated lymphatic tissue in rats from the UCBC + Ad5-LTF group. Our results propose that both approaches of *LTF* gene delivery are efficient for maxillofacial area phlegmon recovery in rats. However, earlier wound healing and better outcomes in cervical lymph node remodeling in the UCBC + Ad5-LTF group, as well as the lack of direct exposure of the viral vector to the organism, which may cause toxic and immunogenic effects, suggest the benefit of cell-mediated gene therapy.

## 1. Introduction

Currently, gene therapy for infection diseases takes 7% from all human gene therapy trials, where HIV infection is the major target [1]. Indeed, the adenoviral (Ad) and adeno-associated viral (AVV) vectors expressing the HIV-1 genes have recently been used as the perspective platforms for HIV-1 vaccine to generate an effective immune response against HIV-1 in humans [2,3]. Another goal of gene therapy for infectious diseases is to temporarily modify the function of epithelial cells to increase the production of naturally occurring antimicrobial molecules. Mainly such gene therapy has been explored as a way of treating burn wounds [4] and diabetic wound infections [5]. However, the efficacy of gene therapy for the treatment of suppurative diseases is still debatable. Abscess of the maxillofacial area is often complicated by a broad range of pathological conditions, including osteomyelitis of the skull, meningitis, brain abscess, and sepsis—the development of these complications as a result of ineffective antimicrobial therapy. Resistance to antibacterial therapy remains the main obstacle and therefore requires the development of novel therapeutical approaches for the treatment of infectious diseases. Thus gene therapy employing recombinant DNA encoding bacteriocidal or bacteriostatic biologically active molecules for enhancing antibiotic therapy may be the promising innovative approach for treatment the suppurative diseases maxillofacial area.

### 1.1. Antimicrobial Gene Therapy

Nowadays, the progress in gene therapy suggests the alternative to antibiotic therapy of infectious diseases is the approach based on the delivery of recombinant genes encoding natural antimicrobial peptides. Host defense antimicrobial peptides as a part of the innate immune system include a variety of biologically active molecules, such as cathelicidins and defensins [6]. These peptides directly interact with viruses, bacteria, and fungi, increasing their membrane permeability and with exotoxins inactivating them. The defensin gene cloned into a retroviral [7], adenoviral [8], and plasmid expression system [9] has shown antibacterial activity in in vitro studies. Adenoviral delivery of LL-37 from the cathelicidin family demonstrated efficacy for the treatment of burn wound infections in rats [4]. Adenoviral vectors carrying genes encoding TNFα, β defensin-3, and LL-37 were tested in mice with experimental latent tuberculosis [10]. Adeno-associated virus (AAV-6) mediated delivery of the b12 human anti-HIV-1 gp120 minibody gene to the lower genital tract of female rhesus macaques provided effective transduction of epithelial cells and prolonged production of b12 minibodies [11]. In a mouse model of ascending infection-related preterm birth, AAV-8 was used to deliver human β-defensin-3 in the cervical mucosa of pregnant mice [12].

### 1.2. Lactoferrin

Lactoferrin (LTF), formerly known as lactotransferrin, is an iron-binding globular glycoprotein that belongs to the transferrin superfamily. Lactoferrin is produced by neutrophils and epithelial secretory cells of mammary, salivary, lacrimal, and bronchial glands. Lactoferrin, as a first-line defense protein, has been reported to have numerous functions, such as immunostimulating, anti-inflammatory, antifungal, antibacterial, antiviral, antiparasitic, and anticarcinogenic activities [13,14,15]. The bacteriostatic effects of lactoferrin are based on the tight binding of Fe^3+^, which is required to synthesize cytochromes and iron-sulfur proteins essential to support bacterial population growth. Lactoferrin can also exert bactericidal effects against many microbes via binding with high affinity to bacterial lipopolysaccharide (LPS), porins, and glycosylated proteins. Clinical application of recombinant lactoferrin to reduce the risk of developing sepsis has been proven in neonates [16,17]. Meanwhile, the rationality in the usage of the lactoferrin gene for therapy of infectious diseases is obscure.

### 1.3. Human Umbilical Cord Blood Cells

The last decade has shown significant progress in applying cell therapy in regenerative medicine. An important role in this strategy belongs to human umbilical cord blood cells (UCBC). The mononuclear fraction of human umbilical cord blood contains different stem cells [18,19,20,21], which suggests using them for the treatment of patients with ischemic, traumatic, and degenerative diseases [22]. The other attractive reason for the use of UCBC for cell therapy is their capability to produce various biologically active molecules with immunostimulating, anti-inflammatory, anti-oxidant, and angiogenic properties [23,24,25,26,27]. The therapeutic power of UCBC may be significantly increased via their genetic modification aimed at temporary production of recombinant therapeutic molecules [28,29,30,31]. Gene modified UCBC may provide delivery of therapeutic transgenes and supply the expression of the recombinant molecules in the target tissue. The therapeutic efficacy of UCBC was tested in a wide variety of pathologic conditions [21,22,32], but UCBC were not examined in the treatment of suppurative diseases.

The development of gene therapy using the adenoviral vector carrying gene encoding LTF for suppurative diseases maxillofacial area is of particular interest. Reviews of randomized controlled trials of genetic therapies revealed a majority of studies using adenoviruses (40%), with 66% reported symptom improvement [33]. The advantages of using the Ad-based vector in comparison with as well commonly used AAV are due to the time peak of *LTF* gene expression in a few hours after injection and transient expression during 3–4 weeks, which is maybe sufficient for the production of recombinant LTF for phlegmon treatment. Major factors limiting the use of adenoviral vectors, such as immunogenicity and toxic effects on recipient cells [34], can be avoided with ex vivo gene therapy. In this study, the efficacy of adenoviral- or UCBC-mediated delivery of the lactoferrin gene for treatment of maxillofacial area phlegmon in a rat model was evaluated.

## 2. Materials and Methods

### 2.1. Gene and Gene-Cell Constructs Preparation

#### 2.1.1. Adenoviral Vectors

Second-generation adenoviral vectors carrying human *LTF* gene and reporter green fluorescent protein (GFP) gene (*gfp*) were generated by the method of a homologous recombination-based on human adenovirus serotype 5 (Ad5) in the Scientific Research Institute of Epidemiology and Microbiology named after N.F. Gamalei (Moscow, Russia), as described previously [35]. AdEasy Adenoviral Vector System (Stratagene, San Diego, CA, USA) was used in order to construct the pAd-Lf plasmid vector containing the genome of the recombinant adenovirus with E1 region deletion, and a transgene expression cassette incorporated instead of it via homologous recombination in *Escherichia coli*. Recombinant adenovirus Ad5-Lf was obtained via transfection of HEK-293 cell lines with the pAd-LTF plasmid construct linearized on the PacI site. Lipofectamine 2000 (Invitrogen, Carlsbad, CA, USA) was used for the transfection, according to the manufacturer’s recommendations. The obtained adenoviruses were purified and concentrated by double ultracentrifugation in a cesium chloride gradient. The titers of Ad5-LTF and Ad5-GFP (2 × 10^10^ PFU/mL and 1.5 × 10^10^ PFU/mL, respectively) were determined by plaque formation assay in HEK-293 cell culture.

#### 2.1.2. Umbilical Cord Blood Mononuclear Cells

All manipulations with umbilical cord blood were performed in accordance with the protocol of the legitimate and ethical standards generally accepted in the stem cell bank of Kazan State Medical University (FS-16-01-001450) on December 26, 2017. UCBC were isolated from fresh umbilical cord blood by the standard procedure of sedimentation onto a density barrier [36]. The obtained cells were transduced with Ad5-LTF or with Ad5-GFP with a total multiplicity of infection equal to 10 (MOI = 10), as described previously [36].

For phlegmon treatment, 0.2 × 10^6^ gene-modified cells (UCBC + Ad5-LTF or UCBC + Ad5-GFP) were prepared in 100 μL of saline per injection. The preparation was injected at five points around the phlegmon so that the total dose per each animal was 1.0 × 10^6^. The selected dose was assigned based on our experience in intravenous [28] and intrathecal [37] administration of gene-modified UCBC.

#### 2.1.3. Evaluation of Transgenes Expression In Vitro

Expression of *LTF* gene in UCBC transduced with Ad5-LTF was evaluated using real-time polymerase chain reaction (RT-PCR) 72 h after incubation of UCBC + Ad5-LTF in six-well plates. Total RNA was isolated from naïve UCBC and UCBC + Ad5-LTF using the TRIzol™ Reagent (Thermo Fisher Scientific, Waltham, MA, USA) according to the manufacturer’s instructions. cDNA was synthesized using six-nucleotide random primers and RevertAid Reverse Transcriptase (Thermo Fisher Scientific). The quantitative analysis of the cDNA samples was performed using a CFX96 thermal cycler (BioRad, Hercules, CA, USA). The reaction mix contained qPCRmix-HS SYBR (Evrogen, Moscow, Russia) and primers (forward: GCCGTAGGAGAAGGAGTGTT and reverse: GCCTGGATACACTGGATGGG). The level of *LTF* gene expression was normalized to the reference gene encoding β-actin using the ΔΔCt (Livak) method of relative quantification [38].

The effectiveness of UCBC transduction with Ad5-GFP was analyzed 72 h after incubation of UCBC + Ad5-GFP in six-well plates. The level of *gfp* reporter gene expression was evaluated by RT-PCR as described above using target-specific primers (forward: AGCAAAGACCCCAACGAGAA and reverse: GGCGGCGGTCACGAA). Production of the green fluorescent protein in the UCBC + Ad5-GFP was examined using fluorescent microscopy.

### 2.2. Modeling and Treatment of Abscesses in Maxillofacial Area

#### 2.2.1. Surgery

In the study, male Wistar rats (7 months old, 250–300 g; Pushchino Laboratory, Pushchino, Russia) were employed. Animals were housed one per cage under standard laboratory conditions, with a 12 h light/dark schedule, 21 ± 3 °C, and ad libitum access to food and water. The animal protocols were conducted in strict compliance with the guidelines established by the Kazan State Medical University Animal Care and Use Committee in 2018 (approval No. 6).

**Figure 1:**
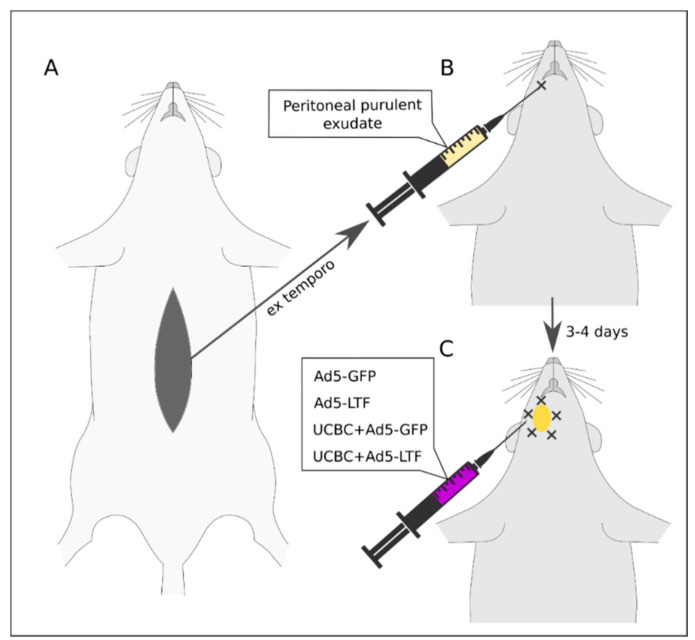
Experimental design. **A**—Acute peritonitis modeling. **B**—Phlegmon modeling by injection of the purulent peritoneal exudate in the molar region of the medial surface of the mandible. **C**—Introduction of one of the preparations after the maturation of the abscess.

All surgical procedures were performed under anesthesia with a mixture of Zoletil 100 (3 mg/kg; Virbac Laboratories, Val de Reuil, France) and Xyla (4.8 mg/kg; Interchemie werken “De Adelaar B.V.”, Venray, Netherlands) administered by intraperitoneal injection and aseptic conditions. The modeling of the maxillofacial area phlegmon included two stages (Figure 1).

(1) Obtaining peritoneal purulent exudate.

Acute peritonitis modeling was performed on the basis of the cecum ligation and puncture method. For this, a midline laparotomy was performed in male rats under the combined anesthesia of Zoletil (80–100 mg/kg) and Xyla (5–15 mg/kg). The cecum was mobilized, the contents of the intestine were pushed towards the dome, and the distal third of the cecum was isolated by ligation with silk thread. Thereafter, the isolated part of the cecum was perforated with a 23 G needle, a through puncture was made in the direction from the mesenteric to the free edge of the cecum, and a small amount (drop) of chyme was squeezed out of the perforation. The cecum was moved into the peritoneal cavity, the tissues of the abdominal wall were sutured in layers, and the skin suture was disinfected. Clinical signs of sepsis began 12 h later as generalized weakness, fever, and decreased motor activity. In the 3–4 days after peritonitis modeling, symptoms of intoxication developed in the rats (apathy, loss of appetite and coordination), repeat laparotomy was performed. Purulent exudate was collected in a syringe. Rats were sacrificed with an intraperitoneal injection of sodium pentobarbital (60 mg/kg; Sigma).

(2) Phlegmon modeling. Ex tempore obtained peritoneal purulent exudate was used for phlegmon modeling. Healthy male rats (*n* = 26) were injected subperiosteally with 0.2 mL of the purulent peritoneal exudate in the molar region of the medial surface of the mandible under general anesthesia. Phlegmon was observed in the parotid and submandibular regions 3–4 days after the injection.

#### 2.2.2. Treatment and Experimental Groups

After incision and drainage of mature abscesses, all animals received antibacterial therapy with ceftriaxone intramuscularly (1000 mg/kg). Following the study, design rats were divided into four groups and were injected subcutaneously at five points around the abscess with 100 μL in each point according to the experimental groups with Ad5-GFP (control group); Ad5-LTF (direct gene therapy group); UCBC + Ad5-GFP (cell therapy group) and UCBC + Ad5-LTF (cell-mediated gene therapy group) (Figure 1 and Table 1). Antibacterial therapy with ceftriaxone was performed in rats from all groups daily to the end of the experiment. In terms of immunogenicity and toxic effect on cells, we have designed the study so that adenoviral vector was included in all (Ad5-GFP, Ad5-LTF, UCBC + Ad5-GFP, UCBC + Ad5-LTF) experimental groups in a way that balanced the possible influence of Ad5 on the phlegmon treatment. In addition, in the Ad5-GFP and UCBC + Ad5-GFP groups, expression of the green fluorescent protein reporter gene was used to assess UCBC homing into cervical lymph nodes and the efficiency of lymph node cell transduction by Ad5-GFP, respectively.

### 2.3. Clinical and Laboratory Examination

#### 2.3.1. Symptomatic Outcomes

The health condition of the animals was assessed daily after surgery to the end of the experiment. Progress in wound healing, vital signs, and time to symptom relief was taken into consideration. Daily monitoring included the main criteria for euthanasia, such as the presence of open wounds, decreases in body temperature, increases of respiratory rate and effort, inability to access food or water, and dehydration (skin pinch test).

#### 2.3.2. Peripheral Blood Analysis

At the end of the experiment, rats were euthanized in accordance with the criteria for euthanasia and humane endpoint. Animals were deeply anesthetized with an intraperitoneal injection of sodium pentobarbital (60 mg/kg; Sigma, St. Louis, MO, USA), and peripheral blood was collected from the right atrium. WBC counts were performed using the Sysmex XP-300 (Sysmex Corporation, Kobe, Japan).

#### 2.3.3. Microbiological Investigation of the Peritoneal Purulent Exudate

The qualitative and quantitative analysis of microbial flora in the peritoneal purulent exudate proposed for use in phlegmon modeling was performed as a specific task on five mature male rats. For bacteriological examinations, 0.1 mL of the ex tempore obtained exudate was sectorally plated on blood agar, which was prepared with the addition of 5% human erythrocyte mass to beef agar. Plates were incubated under aerobic and anaerobic conditions at 37 °C, and colonies were counted after overnight incubation. Bacterial counts were expressed as the number of colony-forming units (CFU) per milliliter of peritoneal exudate fluid. After sufficient growth, the identification of all isolates via the study of proteolytic and saccharolytic properties in accordance with conventional methods was performed. Agar diffusion tests for the antibiotic susceptibility of isolated bacterial strains on Mueller–Hinton agar were carried out in accordance with the guidelines. All strains in the in vitro study were classified as fully susceptible (+), moderately sensitive (±), and insensitive (−).

### 2.4. Histological Investigation of Cervical Lymph Nodes

#### 2.4.1. Morphometric Analysis

For the histological study, animals after blood collection were intracardially perfused with cold 4% paraformaldehyde (Sigma) in phosphate-buffered saline (pH 7.4). The group of cervical lymph nodes from each experimental animal was post-fixed in paraformaldehyde. For morphometric analysis, lymph nodes were embedded in paraffin wax. Total lymph node sections with a 7 μm thickness were stained with hematoxylin and eosin. Areas of lymph node structural components were evaluated using random step morphometric grids and ImageJ software (National Institute of Health (NIH)).

#### 2.4.2. Fluorescent Microscopy Analysis

The post-fixed cervical lymph nodes were cryoprotected in 30% sucrose, and frozen 10 μm thickness sections were prepared. The reporter green fluorescent protein gene expression in the cervical lymph nodes in rats from the Ad5-GFP and UCBC + Ad5-GFP groups was evaluated using fluorescence microscopy. For identification of human UCBC in lymph nodes in rats from the UCBC + Ad5-GFP and UCBC + Ad5-LTF groups, immunofluorescent staining was performed using mouse antibody (Ab) reacting with anti-human nuclear antigen (HNA) (1:150, Millipore, Burlington, MA, USA). Secondary donkey Alexa Fluor 647 conjugated Ab (1:200, Invitrogen) was used for visualization of the primary Ab. Cell nuclear counterstaining was performed with 4′,6-diamidino-2-phenylindole − DAPI (10 μg/mL in PBS, Sigma). Processed sections were embedded in glycerol (GalenoPharm; Saint Petersburg, Russia) and examined with a luminescence microscope (Carl Zeiss Axioscope A1). UCBC (HNA-positive cells) were counted in UCBC + Ad5-GFP and UCBC + Ad5-LTF groups in 10 sections of the cervical lymph node from each rat. Cell counting was performed on digital images in an area of 300 × 240 µm. Average cell counts for each animal were standardized to the area of 1 mm^2^.

### 2.5. Statistical Analyses

Data analysis and visualization were performed using R (version 3.6.3) environment for statistical computing (R Foundation for Statistical Computing, Vienna, Austria). Descriptive statistics for quantitative variables are presented as means ± standard errors and medians (interquartile ranges). The Kruskal–Wallis test and Welch’s *t*-test were used for group comparisons.

## 3. Results

### 3.1. Microbiological Analysis of the Peritoneal Purulent Exudate

The microbial flora of the exudate was presented by the associations of *E. coli* (3 × 10^8^ CFU/mL), *Proteus mirabilis* (2 × 10^8^ CFU/mL), *Enterobacter aerogenes* (2 × 10^8^ CFU/mL), and *Staphylococcus aureus* (2 × 10^3^ CFU/mL). Antibiotic disc diffusion testing of all strains yielded inhibition zone diameters ranging from 6 mm to 74 mm. All isolates were fully susceptible to ceftriaxone, which was chosen for antimicrobial therapy (Table 2).

### 3.2. Molecular Analysis of Gene Modified UCBC In Vitro

**Figure 2:**
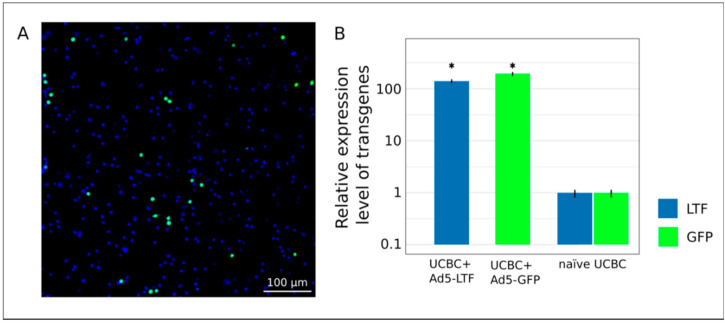
Expression of transgenes in UCBC 72 h after transduction with Ad5-LTF or Ad5-GFP (multiplicity of infection (MOI) = 10). (**A**)—fluorescence microscopy shows a specific green signal in UCBC + Ad5-GFP. Cell nuclei were stained with Hoechst 33342. (**B**)—quantitative analysis of *LTF* and reporter *gfp* genes expression level in UCBC + Ad5-LTF and UCBC + Ad5-GFP in comparison with naïve UCBC. Data are presented as mean ± standard error, * *p* < 0.05.

The efficiency of UCBC transduction with Ad5-GFP and Ad5-LTF was confirmed 72 h after UCBC + Ad5-GFP and UCBC + Ad5-LTF incubation. Using fluorescence microscopy specific green fluorescent signal was detected in the cytoplasm of UCBC + Ad5-GFP (Figure 2A). RT-PCR analysis revealed a significant increase in the level of the *LTF* and reporter *gfp* genes expression in UCBC + Ad5-LTF and UCBC + Ad5-GFP by 125.4 ± 6.7 and 176.8 ± 5.4 times, respectively, when compared to naïve UCBC (*p* < 0.05) (Figure 2B).

### 3.3. Clinical Examination

On the third day after the peritoneal purulent exudate injection, local signs of inflammatory processes in the maxillofacial region were observed in all experimental animals. Clinical findings included edema on the side of the injection, pain reaction on palpation, and food refusal.

**Figure 3:**
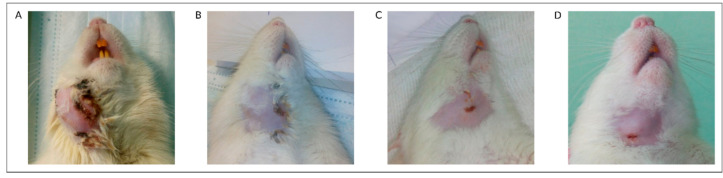
Phlegmon regression. (**A**)—Rat from the control group (Ad5-GFP) on the fourth day of treatment. (**B**)—Rats seven days after injection of UCBC + Ad5-GFP. (**C**,**D**)—Rats from the Ad5-LTF and UCBC + Ad5-LTF groups on the sixth day of treatment.

After the basic treatment of the mature phlegmon, which included incision followed by short-term drainage and continuous antibacterial therapy in control rats (Ad5-GFP), the abscess continued to grow (Figure 3A), and animals developed symptoms that considered to be related to systemic inflammation (complete food refusal, apathy, and lethargy). Animals were euthanized on the fourth or fifth day from the beginning of the treatment according to the ethical rules.

In rats from the Ad5-LTF and UCBC + Ad5-LTF groups, the phase of purulent exudation stopped on the second day of phlegmon treatment. On the third day, granulation tissue appeared in the wound, and on the fourth day, the granulation tissue completely filled the wound. On the fifth to seventh days, the wounds were closed by secondary intention without signs of inflammation (Figure 3C,D). In the experimental group UCBC + Ad5-GFP, the exudation phase stopped on the third day, granulation appeared in the wound on the fourth day, and the postoperative wound was closed by secondary intention on the sixth to seventh days (Figure 3B). Thus, the mean time to wound healing event (WH) in UCBC + Ad5-GFP group was 6.5 days, in Ad5-LTF group—6.5 days, and in UCBC + Ad5-LTF—5.4 days (Table 3). The mean time to euthanasia was 3.8 days due to the development of the signs of systemic inflammation in the Ad5-GFP group.

White blood cell (WBC) count analysis confirmed the positive clinical outcome in rats from the therapeutic groups. In rats from the Ad-GFP group, blood testing revealed leukocytosis (15.6 × 10^9^/L, IQR: 14.1–19.3). Leukocyte counts in the Ad5-LTF (4.9 × 10^9^/L, IQR: 4.0–8.4), UCBC + Ad5-GFP (7.2 × 10^9^/L, IQR: 6.3–9.5) and UCBC + Ad5-LTF (7.0 × 10^9^/L, IQR: 6.0–8.7) groups were significantly lower (*p* = 0.01) in comparison with that in the control group.

### 3.4. Histological Examination of Cervical Lymph Nodes

#### 3.4.1. Morphometric Analysis

**Figure 4:**
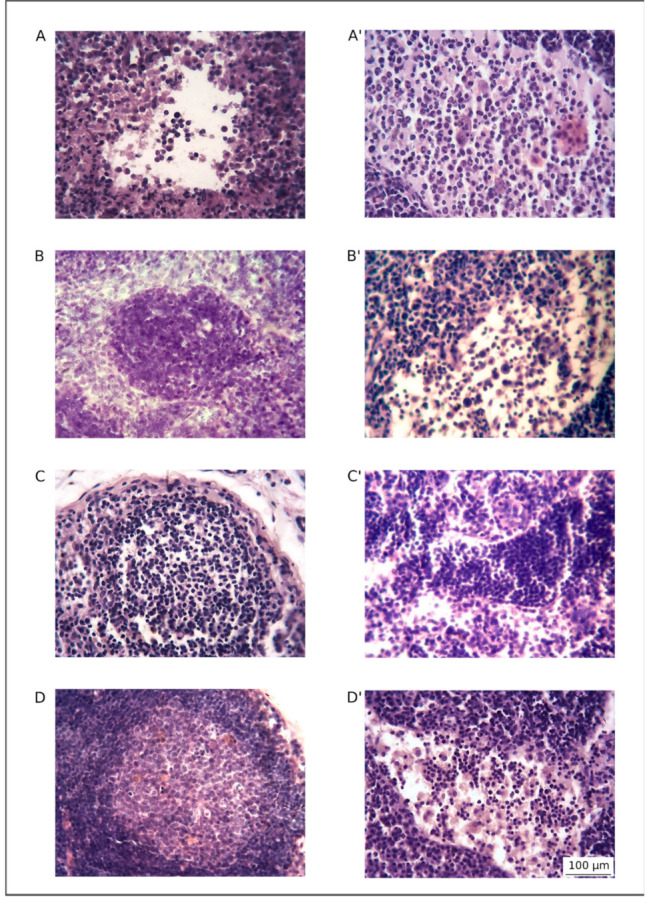
Hematoxylin and eosin staining of rat cervical lymph nodes. Purulent lymphadenitis (**A**) with a focus of destruction of lymphoid tissue (**A’**) in rats from the control Ad5-GFP group. Primary lymphoid follicle without a reactive center (**B**) and monocytoid cells (free macrophages) and lymphocytes in the sinuses (**B’**) in rats from the UCBC + Ad5-GFP group. An attenuated lymphoid follicle (**C**) and sinus lymphocytosis: a large number of lymphocytes in the dilated intermediate sinuses (**C’**) in rats from the Ad5-LTF group. Hyperplastic lymphoid follicle with a large germinal center (**D**) and sinus histiocytosis (**D’**) in rats from the UCBC + Ad5-LTF group.

**Figure 5:**
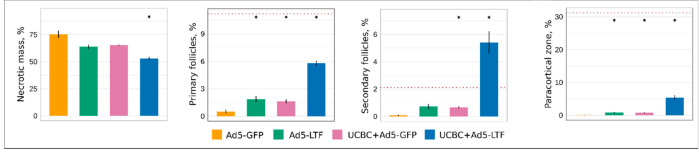
Results of morphometric analysis of rat cervical lymph nodes. Bar plots with error bars illustrate destruction (necrotic mass), preservation (primary follicles and paracortical zone) and activation (secondary follicles) of lymph nodes from the control (Ad5-GFP) and therapeutic (Ad5-LTF, UCBC + Ad5-GFP, and UCBC + Ad5-LTF) groups. Data are presented as mean ± standard error. The dotted lines correspond to the average relative area in intact rats. * *p* < 0.05 in comparison with the Ad5-GFP group.

Histological study revealed different pathological changes in lymph nodes in the experimental groups. Lymph nodes from animals in the Ad5-GFP group demonstrated purulent lymphadenitis with tissue destruction (Figure 4A,A’). Most of the lymph node area was occupied by a necrotic mass with leukocyte infiltration (75.34% ± 3.49%). The unstructured lymphoid tissue presented 21.50% ± 3.33% of the total area (Figure 5).

In the therapeutic groups, more preserved and activated lymphatic tissue was documented. Decrease of the necrotic mass area to 65.23% ± 0.63% in the UCBC + Ad5-GFP group (Figure 4B,B’) and to 63.82% ± 1.86% in the Ad5-LTF group (Figure 4C,C’) was observed (Figure 5). In the preserved lymphoid tissue, structural components (lymphoid follicles and sinuses) were observed. Lymphoid follicles were predominant without germinal centers or attenuated type. Morphological changes in the UCBC + Ad5-LTF group showed a significant decrease in the area of necrotic mass to 52.97% ± 1.58% and the preservation of structured lymph node tissue. Follicular and paracortical hyperplasia, as well as sinus histiocytosis, were observed (Figure 4D,D’). The follicles were enlarged, with large light germinal centers, in which the mitotic activity was increased, and macrophages appeared.

#### 3.4.2. Homing UCBC in the Cervical Lymph Nodes

**Figure 6:**
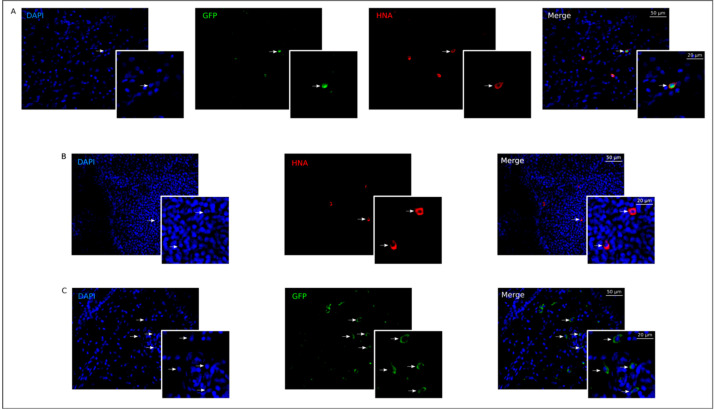
Homing UCBC and expression of reporter GFP in rat cervical lymph nodes. Immunofluorescent staining of lymph nodes with an antibody (Ab) to the human nuclear antigen (HNA) in UCBC + Ad5-GFP group (**A**) and in UCBC + Ad5-LTF group (**B**). Arrows indicate HNA-positive cells (red) 7 days after UCBC injection, a green signal corresponds GFP. (**C**)—Fluorescent microscopy of a lymph node from the Ad5-GFP group. Arrows indicate specific fluorescent signal of GFP in host lymphatic cells five days after injection of Ad5 carrying reporter gene, encoding GFP. Cell nuclei were counterstained with DAPI (blue) Scale bar in (A–C) = 50 μm; scale bar in the insert = 20 μm.

An antibody to the human nuclear antigen (HNA) was used for the identification of human UCBC in the rat cervical lymph nodes seven days after injection of UCBC + Ad5-GFP or UCBC + Ad5-LTF. A few HNA-positive UCBC were found in both UCBC + Ad5-GFP (28.1 ± 2.9 cells/mm^2^) and UCBC + Ad5-LTF (34.9 ± 7.1 cells/mm^2^) groups (*p* = 0.42) (Figure 6A,B). In the UCBC + Ad5-GFP group, HNA-positive cells had a specific green signal (Figure 6A). This observation and the data on the similar level of transduction UCBC with Ad5-GFP or Ad5-LTF (shown above) presents the evidence that UCBC, after subcutaneous injection at the sites of the maxillofacial area abscesses, may migrate into the regional cervical lymph nodes and produce recombinant proteins.

#### 3.4.3. Fluorescent Analysis of Reporter *gfp* Gene Expression in the Cells of Cervical Lymph Nodes

Fluorescent microscopy of lymph nodes in rats from the Ad5-GFP group revealed GFP-positive cells in the lymphatic follicles and sinuses (Figure 6C). In the green channel, GFP-positive cells demonstrated specific green fluorescence located in the cytoplasm. This data suggest that Ad5 carrying the *gfp* reporter gene with lymph flow diffuse into the regional lymph node and transduce lymphatic cells, which were capable of efficiently producing GFP.

## 4. Discussion

In practice, recombinant lactoferrin in combination with antibiotics is used for the treatment of neonatal sepsis [39] and for modification of the fecal microbiome in infants [40].

In gene therapy, LTF is used as a ligand in a vector system. Intravenous administration of LTF-modified nanoparticles have been used for delivery of human glial cell line-derived neurotrophic factor gene [41]. LTF, by binding to its receptors, provides the ability to cross the blood-brain barrier (BBB) and brain-targeting of the nanoparticles carrying the therapeutic gene. Conjugation of LTF to 3-diaminobutyric polypropylenimine dendrimer carrying plasmids encoding for TNFα, TRAIL, or IL-12 improved its transfection and therapeutic efficacy in prostate cancer cells [42].

It is known that LTF is rapidly cleared from the circulation (~12.6 min half-life for recombinant human LTF in rats), which limits its therapeutic potential [43]. However, LTF mRNA is a fairly stable molecule, with a half-life of between 8 and 9 h [44]. Thus, in the study for stable and prolonged production of recombinant LTF, we suggested the use of the *LTF* gene for treatment of maxillofacial area abscesses in a rat model.

Direct adenoviral- and UCBC-mediated *LTF* gene delivery was examined. The analysis of the obtained results demonstrated the clinical, laboratory, and morphological improvement in the following order: UCBC + Ad5-LTF ˃ Ad5-LTF ˃ UCBC + Ad5-GFP. In the control group (Ad5-GFP), the rats who received antibacterial therapy with ceftriaxone did not recover and were euthanized on the third to fourth day after phlegmon sanitation. In vitro estimated peritoneal purulent exudate microflora sensitivity to ceftriaxone did not approve its efficacy in vivo. It is possible that unidentified bacteria in the exudate and the joined pathogenic microflora during the maturation of the phlegmon can also be the reasons for the insensitivity of therapy with ceftriaxone. Meanwhile, GFP-positive cell observation in the cervical lymph nodes after Ad5-GFP injection suggests the Ad5-LTF pattern of diffusion and lymphatic cell transduction. Thus, the positive effect (phlegmon regression on 6.6 days, lymph node tissue preservation, and the normal number of white blood cells) of direct adenoviral *LTF* gene delivery may be due to transduction of the host lymphatic cells and local production of the recombinant LTF.

The best therapeutic outcome was demonstrated in rats treated with UCBC + Ad5-LTF, which all recovered with wound healing on day 5.6 by secondary intention with normal leukocyte counts and positive lymph node remodeling. Cell therapy with UCBC showed a less pronounced effect on the outcomes of phlegmon (day 6.5). Meanwhile, the time to symptom relief and wound healing indicate a positive influence of UCBC. Possibly, UCBC after subcutaneous injection migrated into the lymph node and stimulated host immune cells. Thus, treatment with UCBC + Ad5-LTF had synergistic effects of UCBC and recombinant LTF on the recovery of rats with maxillofacial abscesses.

To obtain the reliable effects of *LTF* gene therapy, the following approaches can be considered to overcome the limitations in the current study. First, the local delivery of recombinant LTF might be used. The potential use of human recombinant LTF is achievable and is used for therapy of the broad spectrum of conditions, including infection diseases as a nutraceutical [15]. Second, the studies on larger-sized animals such as pigs [5,45] would be necessary to explore the potential therapeutic outcomes. These critical considerations make up some important limitations before the translation of the direct or cell-mediated *LTF* gene therapy for the treatment of maxillofacial area abscess in patients.

The wide usage of UCBC in practice is obscure. Although UCBC are characterized as cells with low immunogenicity and are used in the numerous clinical trials for the treatment of somatic diseases, allogeneic infusion of UCBC may cause anticipated side effects, such as an immune response in recipient, graft versus host disease, or transformation of the umbilical cord blood stem cells into tumor cells. Moreover, UCBC therapy currently has ethical and legal issues, and a relatively small amount of UCBC from one donor for a single infusion and the impossibility of repeated infusions of cells from the same donor imposes a severe limitation for extensive application of UCBC for cell and cell-mediated gene therapy in adults. The further development of the strategy to use leucocytes for addressed delivery of transgenes and for local production of the therapeutic molecules is aimed at replacement of UCBC on autologous leucocytes obtained from peripheral blood for production of recombinant LTF following our original method preparation of genetically-enriched autologous leucoconcentrates for personalized ex vivo gene therapy [46]. In our recent study, we proposed to use genetically-enriched autologous leukoconcentrate for temporary, simultaneous production of three recombinant neurotrophic factors (vascular endothelial growth factor, glial cell-line derived neurotrophic factor, and neural cell adhesion molecule) for stimulation regeneration in the CNS [46]. Based on the present data, we theorized that *LTF* gene-enriched autologous leukoconcentrate might be an effective antimicrobial drug for personalized therapy. Enrichment of the autologous leukoconcentrate simultaneously with genes encoding lactoferrin, cathelicidins, and defensins may increase the benefit of treatment of infectious diseases in patients.

## 5. Conclusions

Adenoviral- and UCBC-mediated lactoferrin gene delivery for gene therapy of the maxillofacial area abscesses in a rat model was effective and was characterized by wound healing by secondary intention, normal blood count, and positive lymph node remodeling. These data propose the possibility to use adenoviral vectors carrying *LTF* gene to prepare genetically-enriched leukoconcentrate obtained from peripheral blood of the patient for antimicrobial therapy.

## Figures and Tables

**Table 1:** Experimental groups of animals.

Groups	Preparations for Animal Treatment	Number of Animals
Ad5-GFP	Ad5 carrying *gfp* (1 × 10^8^ PFU) in 0.1 mL of 0.9% NaCl	5
Ad5-LTF	Ad5 carrying *LTF* (1 × 10^8^ PFU) in 0.1 mL of 0.9% NaCl	8
UCBC + Ad5-GFP	0.2 × 10^6^ UCBC transduced with Ad5 carrying *gfp* in 0.1 mL of 0.9% NaCl	8
UCBC + Ad5-LTF	0.2 × 10^6^ UCBC transduced with Ad5 carrying *LTF* in 0.1 mL of 0.9% NaCl	5

Ad5: adenoviral vector GFP: green fluorescent protein; LTF: lactoferrin UCBC: umbilical cord blood mononuclear cells.

**Table 2:** Antibiotic susceptibility of strains of isolated bacteria in vitro.

Antibacterial Agent	*E. coli*	*S. aureus*	*E. aerogenes*	*P. mirabilis*
Oxacillin	+	±	±	±
Erythromycin	+	±	+	+
Vancomycin	±	+	+	+
Gentamicin	±	–	–	+
Ciprofloxacin	±	+	+	±
Furadonin	–	–	+	–
Cefixime	+	–	+	–
Ampicillin	–	–	–	–
Chloramphenicol	±	+	–	+
Rifampicin	+	±	+	–
Trimethoprim	±	–	±	+
Tetracycline	+	–	+	±
Ceftriaxone	+	+	+	+

**Table 3:** Cumulative number of wound healing (WH) and euthanasia (EU) events in experimental groups.

Days	Ad5-GFP (*n* = 5)		UCBC + Ad5-GFP (*n* = 8)		Ad5-LTF (*n* = 8)		UCBC + Ad5-LTF (*n* = 5)	
	WH	EU	WH	EU	WH	EU	WH	EU
1	0	0	0	0	0	0	0	0
2	0	0	0	0	0	0	0	0
3	0	1	0	0	0	0	0	0
4	0	5	0	0	0	0	0	0
5	0	5	0	0	0	0	3	0
6	0	5	4	0	4	0	2	0
7	0	5	8	8	8	8	5	5

## Data Availability

The data presented in this study are included in this published article.
